# Membrane-anchored human Rab GTPases directly mediate membrane tethering *in vitro*

**DOI:** 10.1242/bio.20149340

**Published:** 2014-10-31

**Authors:** Naoki Tamura, Joji Mima

**Affiliations:** Institute for Protein Research, Osaka University, Suita, Osaka 565-0871, Japan

**Keywords:** Rab GTPase, Liposome, Membrane tethering, Membrane traffic, Reconstitution

## Abstract

Rab GTPases are master regulators of eukaryotic endomembrane systems, particularly functioning in membrane tethering to confer the directionality of intracellular membrane trafficking. However, how exactly Rab GTPases themselves act upon membrane tethering processes has remained enigmatic. Here, we thoroughly tested seven purified Rab GTPases in human, which localize at the various representative organelles, for their capacity to support membrane tethering *in vitro*. Strikingly, we found that three specific human Rabs (endoplasmic reticulum/Golgi Rab2a, early endosomal Rab5a, and late endosomal/lysosomal Rab7a) strongly accelerated membrane aggregation of synthetic liposomes even in the absence of any additional components, such as classical tethers, tethering factors, and Rab effectors. This Rab-induced membrane aggregation was a reversible membrane tethering reaction that can be strictly controlled by the membrane recruitment of Rab proteins on both apposing membranes. Thus, our current reconstitution studies establish that membrane-anchored human Rab GTPases are an essential tethering factor to directly mediate membrane tethering events.

## INTRODUCTION

Eukaryotic cells organize and maintain the complex but highly specific secretory and endocytic trafficking pathways to deliver correct sets of cargo molecules towards their various subcellular organelles and plasma membranes (Bonifacino and Glick, 2004). These membrane trafficking events are temporally and spatially regulated by a variety of key protein components, including SNARE proteins (Jahn and Scheller, 2006), SNARE-binding cofactors such as Sec1/Munc18 proteins (Rizo and Südhof, 2012), Rab GTPases (Stenmark, 2009), and Rab-interacting effector proteins (Grosshans et al., 2006). Membrane tethering, the first contact of organelles and transport vesicles before membrane docking and fusion, is a critical step to control the directionality of membrane traffic and has been proposed to be mediated by Rab GTPases and Rab-effector proteins (Yu and Hughson, 2010). However, it has still remained ambiguous how Rabs and their effectors directly act on membrane tethering, although several reconstitution studies have reported that yeast endosomal Rab GTPases and the HOPS complex, a Rab effector at yeast vacuoles, had the intrinsic capacity to tether liposomal membranes (Lo et al., 2012; Stroupe et al., 2009; Hickey and Wickner, 2010; Wickner, 2010). In this study, to address the issue, we thoroughly investigated seven representative Rab GTPases in human, which localize at the distinct subcellular compartments, by analyzing their inherent potency to directly promote membrane tethering *in vitro*.

## RESULTS AND DISCUSSION

**Fig. 1.**
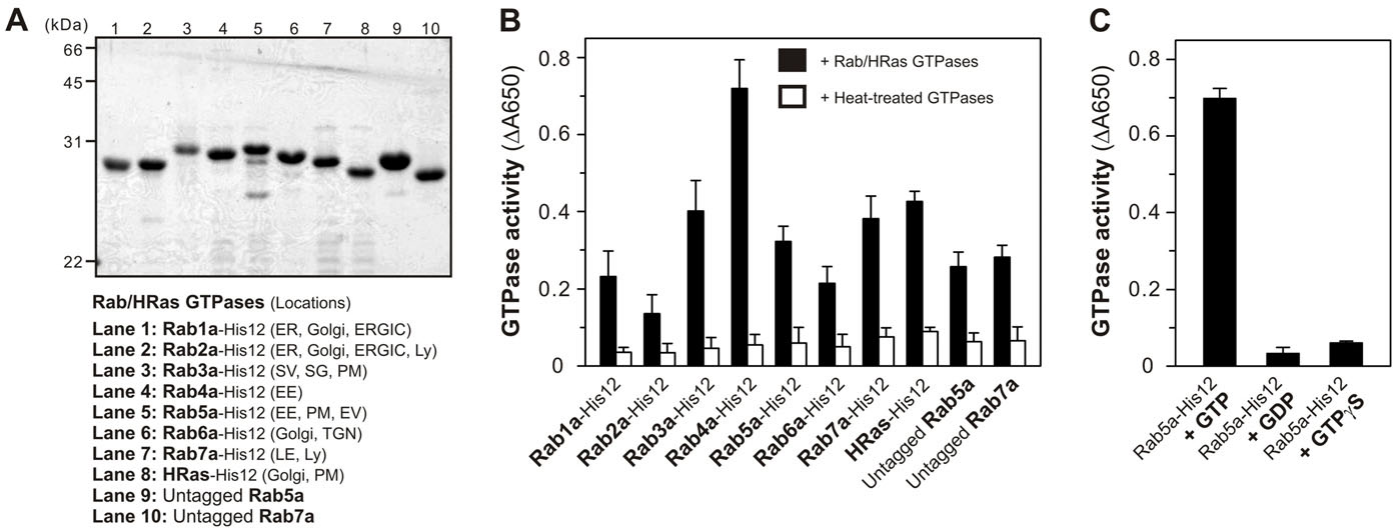
GTP-hydrolysis activities of purified human Rab GTPases. (A) The Coomassie Blue-stained gel of purified recombinant human Rab and HRas GTPases used in this study. The subcellular locations are indicated (ER, endoplasmic reticulum; Golgi; ERGIC, ER-Golgi intermediate compartment; Ly, lysosome; SV, secretory vesicle; SG, secretory granule; PM, plasma membrane; EE, early endosome; EV, endocytic vesicle; TGN, trans-Golgi network; and LE, late endosome). (B) All the purified recombinant Rab and HRas proteins had the intrinsic GTP-hydrolysis activities. GTPase activities of Rab-His12 proteins, HRas-His12, and untagged Rab proteins (4 µM final for each) were assayed using a Malachite Green-based reagent to quantify released free phosphate molecules, by measuring the absorbance at 650 nm (black bars). For a control, the same GTPase-activity assays were also performed with denatured Rab and HRas proteins that had been heat-treated at 100°C for 15 min (white bars). (C) Purified recombinant Rab5a-His12 specifically hydrolyses GTP. GTPase activity of Rab5a-His12 (6 µM final) was assayed as in panel B, but in the presence of GTP (1 mM), GDP (1 mM), or GTPγS (1 mM), where indicated.

**Fig. 2.**
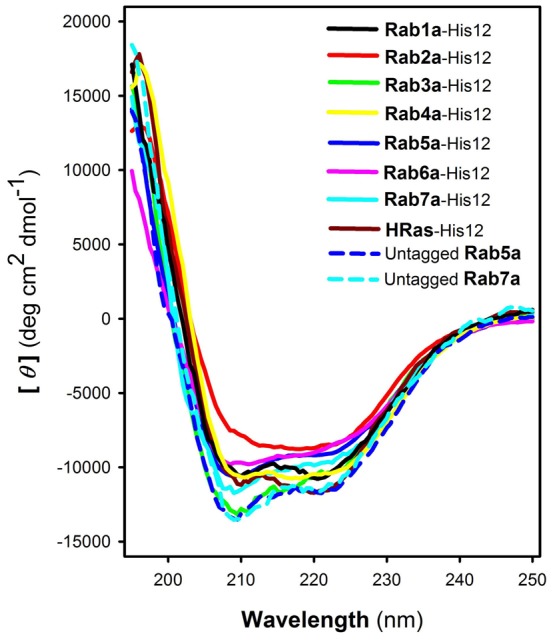
CD spectra of purified human Rab GTPases. Far-UV CD spectra of Rab1a-His12 (black), Rab2a-His12 (red), Rab3a-His12 (green), Rab4a-His12 (yellow), Rab5a-His12 (blue), Rab6a-His12 (pink), Rab7a-His12 (cyan), HRas-His12 (brown), untagged Rab5a (blue dashed line), and untagged Rab7a (cyan dashed line), in HN150 (20 mM Hepes-NaOH, pH 7.4, 150 mM NaCl) containing glycerol (10%), MgCl_2_ (5 mM), and DTT (1 mM).

Rab GTPases are typically post-translationally modified by an isoprenyl lipid group at their C-terminal cysteine residues, which is required for membrane association of Rabs (Hutagalung and Novick, 2011). To mimic the membrane-bound state of native Rabs bearing the lipid anchor, the seven selected human Rabs were purified as the C-terminal polyhistidine-tagged forms (Rab-His12 proteins) that can be attached to liposome membranes bearing a DOGS-NTA lipid (1,2-dioleoyl-*sn*-glycero-3-{[*N*-(5-amino-1-carboxypentyl)iminodiacetic acid]-succinyl}) (Fig. 1A, lanes 1–7). For a negative control, we also purified the His12-tagged form of human HRas, which is a similar Ras-family GTPase with a C-terminal lipid anchor but not functionally related to membrane tethering events (Fig. 1A, lane 8). All the purified Rab-His12 and HRas-His12 proteins retained their intrinsic GTP-hydrolysis activities, specifically converting GTP to GDP and a free phosphate group (Fig. 1B,C). In addition, we further characterized the purified Rab proteins by circular dichroism (CD) spectroscopy (Fig. 2). All the six Rab-His12 proteins tested, except Rab2a-His12, had similar far-UV CD spectra (Fig. 2) and comparable secondary structure contents which were estimated from the CD spectra using a K2D3 program (Table 1) (Louis-Jeune et al., 2012). These biochemical properties support that those six Rab-His12 proteins are a well-folded protein that indeed has the capacity to bind and hydrolyze a guanine nucleotide. However, as Rab2a-His12 showed significant differences from the other Rab-His12 proteins in the CD spectra and predicted secondary structure contents, it should be noted that our preparation of Rab2a-His12 likely contained some denatured or partially-denatured proteins.

**Table 1.**
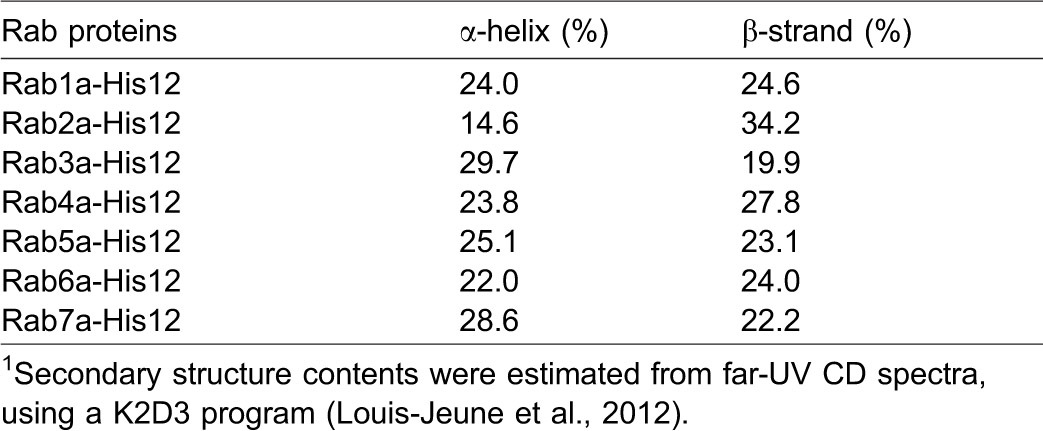
Predicted secondary structure contents of purified Rab-His12 proteins^1^

### Three selective Rab GTPases specifically promote robust liposome aggregation

**Fig. 3.**
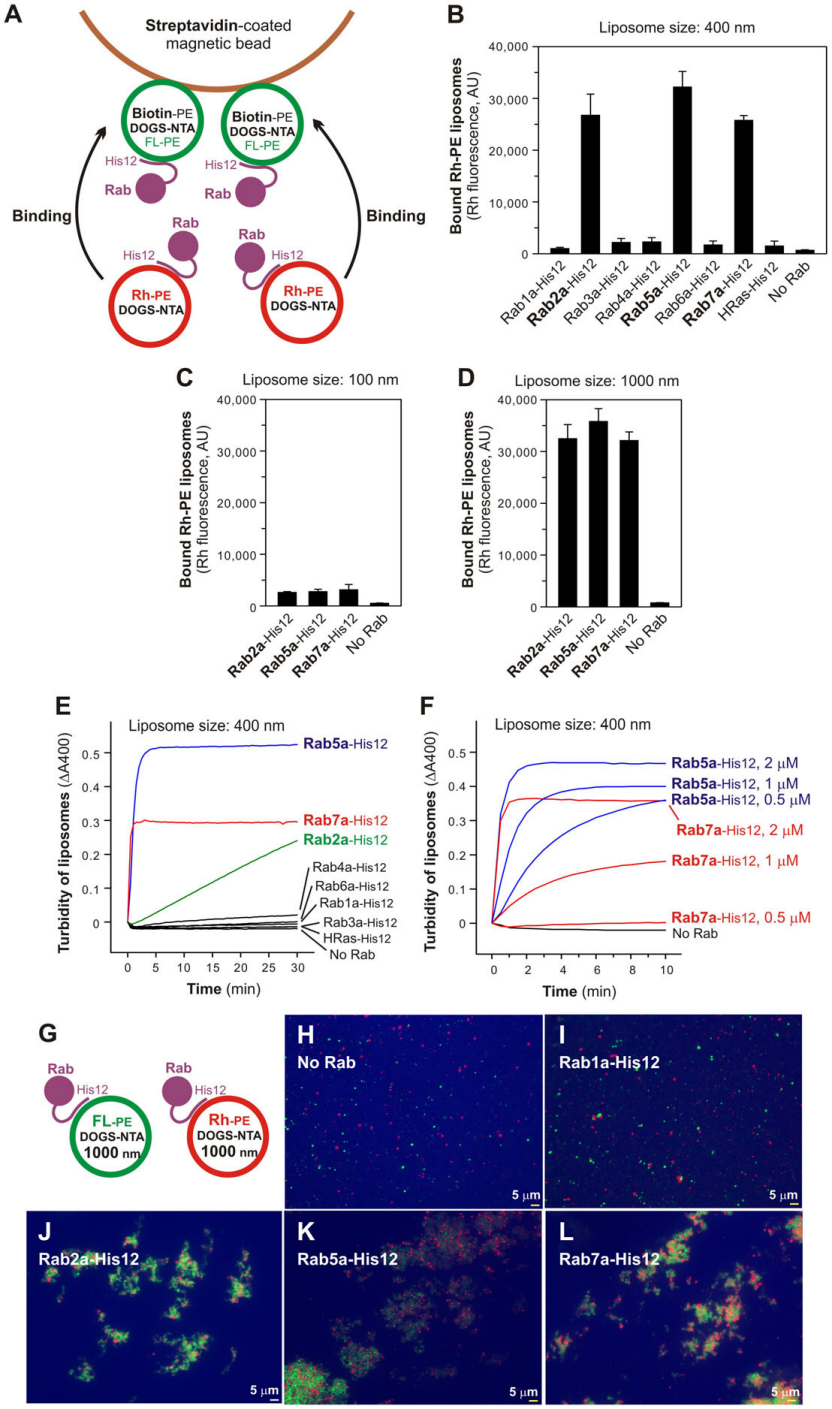
Three human Rab GTPases are specific proteins to drive liposome aggregation. (A) Schematic representation of the liposome aggregation assay using streptavidin-coated beads, two types of liposomes bearing either biotin-PE/DOGS-NTA/FL-PE or Rh-PE/DOGS-NTA, and purified Rab-His12 proteins. (B–D) Rab2a, Rab5a, and Rab7a promote robust liposome aggregation. The Rh-labeled liposomes (1.5 mM lipids) were mixed with the biotin-labeled liposomes (1.8 mM lipids), Rab-His12 proteins (4 µM), and streptavidin beads, and incubated (30°C, 2 hours). The Rh-labeled liposomes co-isolated with streptavidin beads were analyzed by measuring the Rh fluorescence. Liposomes were prepared by extrusion through 400 nm (B), 100 nm (C), or 1000 nm (D) filters. AU, arbitrary units. (E,F) Kinetics of Rab-induced liposome aggregation. To monitor turbidity changes of liposome suspensions with Rabs, liposomes (1.3 mM lipids) were mixed with Rab-His12 proteins [2 µM (E), 0.5–2 µM (F)], followed by measuring the absorbance at 400 nm. (G–L) Rab2a, Rab5a, and Rab7a induce the formation of massive liposome clusters. As represented in panel G, the FL-PE liposomes (1.8 mM lipids) and Rh-PE liposomes (1.5 mM lipids) were mixed without Rabs (H) or with Rab1a-His12 (I), Rab2a-His12 (J), Rab5a-His12 (K), and Rab7a-His12 (L) (4 µM each). After incubation (30°C, 2 hours), fluorescence images of the liposome suspensions were obtained. Scale bars: 5 µm.

Using purified Rab-His12 proteins, two types of liposomes that bore DOGS-NTA and either biotin-labeled phosphatidylethanolamine (biotin-PE) or rhodamine-labeled PE (Rh-PE), and streptavidin-coated beads, we developed an *in vitro* assay to test whether membrane-bound Rabs promote liposome aggregation (Fig. 3A). Reaction mixtures containing those two distinct liposomes decorated with Rab-His12 proteins were incubated with streptavidin beads to isolate the biotin-PE liposomes, followed by measuring Rh fluorescence for quantifying the amounts of the Rh-PE liposomes co-isolated with the biotin-PE liposomes (Fig. 3A). Strikingly, three specific Rabs (Rab2a at endoplasmic reticulum (ER)/Golgi, Rab5a at early endosomes, and Rab7a at late endosomes or lysosomes) supported stable association of the Rh-PE liposomes with the biotin-PE liposomes, whereas the other four Rabs (Rab1a, Rab3a, Rab4a, and Rab6a) and HRas had little effect on assemblies of these liposomes (Fig. 3B). However, even those three active Rabs (Rab2a, Rab5a, and Rab7a) were not able to initiate efficient assemblies of highly curved, small liposomes prepared by extrusion through a 100-nm pore filter (Fig. 3C), in contrast to relatively large-size liposomes extruded through a 400-nm or 1000-nm filter (Fig. 3B,D). This reflects that the robust activities for the three Rabs to promote liposome assemblies are dependent on the size of liposomes used. Membrane tethering of small highly-curved vesicles may require the other additional factors that sense membrane curvature, as previously reported for human golgin GMAP-210, the Golgi-associated coiled-coil protein which contains an ALPS (amphipathic lipid-packing sensor) motif (Drin et al., 2007; Drin et al., 2008).

To further characterize the Rab-induced liposome assemblies, we employed turbidity assays of liposome suspensions in the presence of Rab proteins (Fig. 3E,F). In accord with the results in streptavidin-bead assays (Fig. 3B), the same three specific Rabs (Rab2a, Rab5a, and Rab7a) caused robust increases in the turbidity of liposome suspensions (Fig. 3E). In particular, Rab5a and Rab7a strongly accelerated the initial rates of the turbidity increases (Fig. 3E), which thoroughly depend on the Rab concentrations added (Fig. 3F). Furthermore, fluorescence microscopic observations of the Rab-decorated liposomes revealed that those three active Rabs induced the formation of huge clusters of aggregated liposomes (Fig. 3G–L). Thus, the current three *in vitro* analyses, including the streptavidin-bead assay (Fig. 3B–D), turbidity assay (Fig. 3E,F), and fluorescent microscopy (Fig. 3G–L), demonstrate that specific human Rab GTPases can mediate liposome aggregation, even when their specific Rab effectors and/or other tethering factors are not present. These results are partially consistent with the recent study reporting the intrinsic liposome-tethering activity of the yeast Rab5 ortholog Vps21p, but not the Rab7 ortholog Ypt7p (Lo et al., 2012).

### Exogenously added guanine nucleotides have no effect on Rab-induced membrane aggregation

**Fig. 4.**
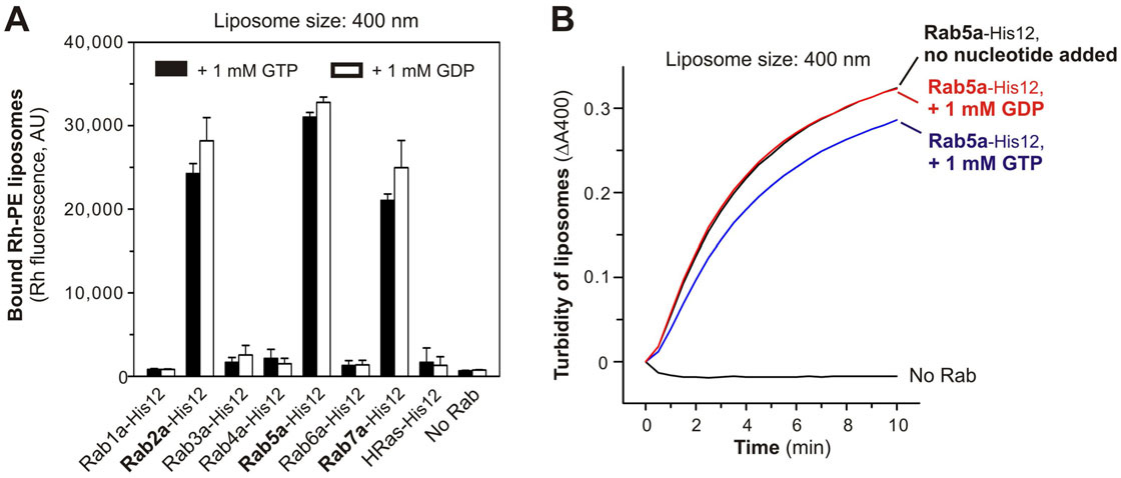
Rab-induced liposome aggregation in the presence of exogenous guanine nucleotides. (A) Addition of exogenous guanine nucleotides has no effect on Rab-induced liposome aggregation. Liposome aggregation assays were employed as in Fig. 3B, but in the presence of 1 mM GTP or GDP. (B) Turbidity changes of liposome suspensions were assayed for Rab5a-His12 (0.5 µM) as in Fig. 3E,F, but in the presence of GTP/GDP.

In general, Rab GTPases are thought to be activated in their GTP-bound states and thereby interact with their specific Rab effectors for the downstream functions, including membrane tethering and docking (Grosshans et al., 2006; Stenmark, 2009; Hutagalung and Novick, 2011). Moreover, the prior *in vitro* analyses of yeast Rabs indicated that the tethering activity of the yeast Rab5/Vps21p relied on its GTP-loaded form (Lo et al., 2012). In this context, we asked whether guanine nucleotides are an essential component in *in vitro* membrane aggregation mediated by human Rabs (Fig. 4), even though we had observed that at least three Rabs (Rab2a, Rab5a, and Rab7a) initiated robust liposome aggregation without adding guanine nucleotides (Fig. 3). Notably, when GTP and GDP were exogenously added in the streptavidin bead assays and turbidity assays, these nucleotides had no significant effect on the Rab-dependent liposome aggregation reactions (Fig. 4A,B). Under these current experimental conditions, we observed that added GTP did not restore or further stimulate the capacity of the human Rab GTPases to promote membrane aggregation, and also that GDP addition had no inhibitory effect on liposome aggregation reactions by the three active Rab GTPases, Rab2a, Rab5a, and Rab7a (Fig. 4A,B). Further reconstitution studies with guanine nucleotide-preloaded Rabs, the Rab-specific guanine nucleotide exchange factors, and the Rab GTPase-activating proteins will be required to more thoroughly assess the GTP/GDP-dependence of Rab-mediated membrane aggregation.

### Membrane-anchored Rab proteins specifically mediate reversible membrane tethering reactions

**Fig. 5.**
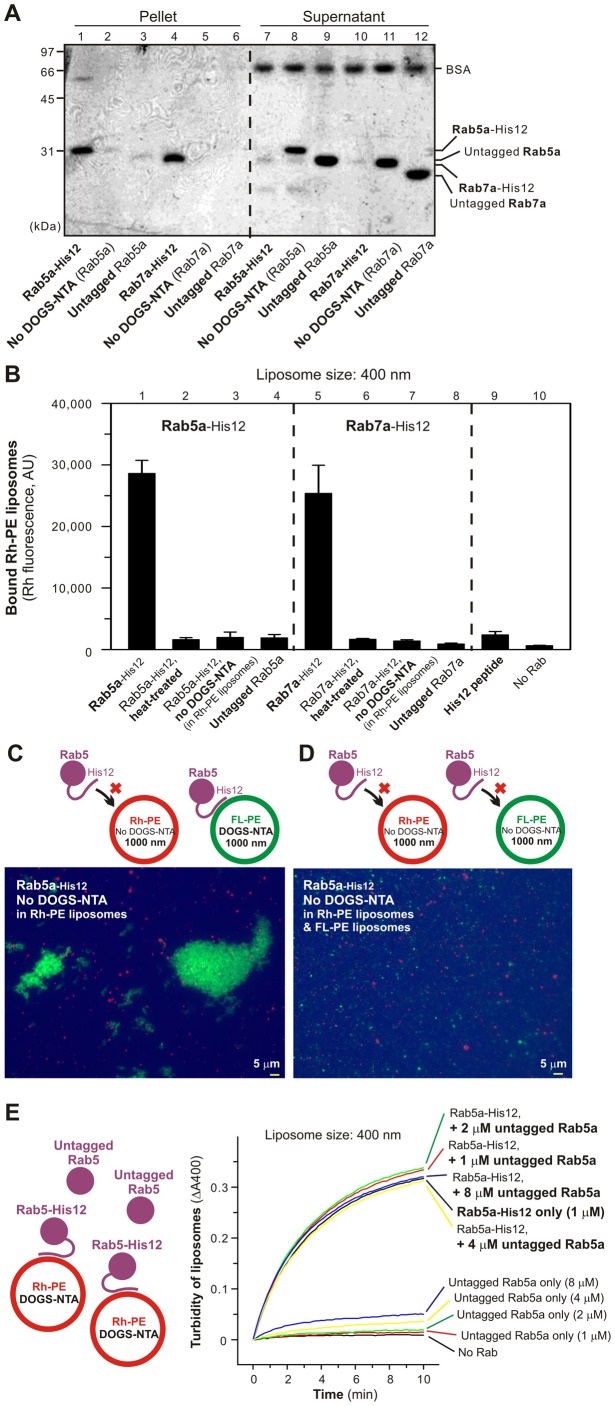
Rab-mediated liposome aggregation requires membrane recruitment of Rabs on both apposing membranes. (A) Liposome co-sedimentation assays. The Rh-PE liposomes (1.5 mM lipids) were mixed with Rab proteins (4 µM), incubated (30°C, 2 hours), centrifuged, and analyzed by SDS-PAGE and Coomassie Blue staining. The Rh-PE liposomes lacking DOGS-NTA were used instead where indicated (no DOGS-NTA, lanes 2, 5, 8, and 11). (B) Liposome aggregation was assayed as in Fig. 3B, with Rab5a-His12, Rab7a-His12, heat-treated Rab-His12 proteins (lanes 2 and 6), untagged Rabs (lanes 4 and 8), and a His12 peptide (lane 9). The Rh-PE liposomes lacking DOGS-NTA was used instead where indicated (no DOGS-NTA in Rh-PE liposomes, lanes 3 and 7). (C,D) Fluorescence microscopy was performed as in Fig. 3H–L, with Rab5a-His12, the Rh-PE liposomes lacking DOGS-NTA, and the FL-PE liposomes that bear DOGS-NTA (C) or not (D). (E) Addition of untagged Rab5a does not competitively block Rab5a-induced liposome aggregation. Turbidity changes of liposome suspensions (1.0 mM lipids) were assayed for Rab5a-His12 (1.0 µM) as in Fig. 3E,F, but in the presence of untagged Rab5a (1–8 µM). Scale bars: 5 µm.

We next tested whether membrane attachment of Rab proteins is indeed indispensable for their specific function to cause membrane aggregation (Fig. 5). Liposome co-sedimentation assays confirmed that Rab-His12 proteins were stably bound to the DOGS-NTA-containing liposomes (Fig. 5A, lanes 1 and 4) and that the membrane attachment of Rabs was fully abolished when used the liposomes lacking DOGS-NTA or the untagged Rabs without a C-terminal His12 tag instead (Fig. 5A, lanes 2, 3, 5, and 6). Strikingly, Rab5a and Rab7a completely lost their potency to initiate liposome aggregation under those conditions where Rabs no longer stably associated with liposomal membranes (Fig. 5B–D). Moreover, we found that Rab5a and Rab7a had to be anchored on both, not either one, of two opposing liposomal membranes for driving membrane aggregation (Fig. 5B, lanes 3 and 7; Fig. 5C), suggesting that Rab-induced membrane aggregation is promoted by *trans*-Rab protein assemblies on apposed membranes. Next, to test whether Rab-mediated liposome aggregation can be competitively blocked by addition of untagged Rab proteins, which have no C-terminal His12 tag for membrane attachment but may be able to associate with membrane-anchored Rab-His12 proteins, we employed the turbidity assays in the presence of both Rab5a-His12 and untagged Rab5a (Fig. 5E). However, even at 8-fold molar excess of untagged Rab5a over Rab5a-His12, the soluble untagged Rab5a protein had little inhibitory effect on Rab5a-mediated liposome aggregation (Fig. 5E). This may reflect that membrane-anchored Rab5a exclusively recognize and assemble in *trans* with Rab5a on opposing membranes destined to tether, not membrane-detached soluble Rab5a, thereby conferring specific membrane tethering events.

**Fig. 6.**
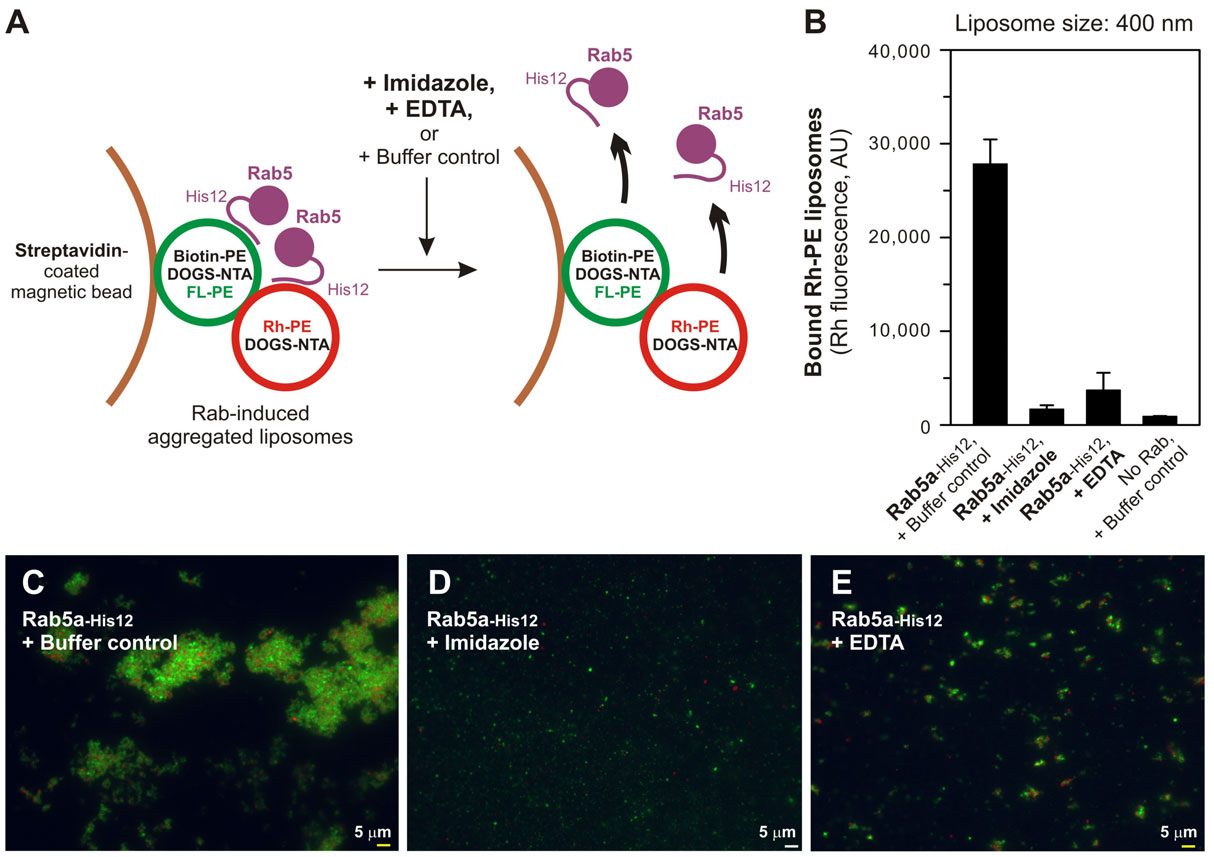
Rab-mediated liposome aggregation is a reversible membrane tethering reaction. (A) Schematic representation of the liposome aggregation assays in panel B, in which imidazole or EDTA was supplemented to detach Rab5a-His12 from DOGS-NTA-bearing liposomes. (B) Addition of imidazole and EDTA causes the dissociation of Rab-induced liposome aggregates. After the biotin-PE liposomes and Rh-PE liposomes were mixed and incubated (30°C, 2 hours) with Rab5a-His12 and streptavidin beads, the liposome suspensions were supplemented with the buffer control, imidazole (500 mM), or EDTA (20 mM), further incubated (30°C, 2 hours), and analyzed as in Fig. 3B. (C–E) Rab-induced massive liposome clusters were disrupted by addition of imidazole or EDTA. Liposome suspensions were incubated with the buffer control (C), imidazole (D), and EDTA (E) as in panel B, but without streptavidin beads. Fluorescence images were obtained as in Fig. 3H–L. Scale bars: 5 µm.

We then asked whether the liposome aggregation reactions mediated by membrane-anchored Rab proteins are indeed a reversible reaction, like physiological membrane tethering events (Ungermann et al., 1998). To address this, the pre-formed Rab5a-mediated liposome aggregates were further incubated with imidazole and EDTA, which lead to dissociation of Rab5a-His12 from DOGS-NTA-bearing liposomes, and then tested by the streptavidin-bead assay (Fig. 6A,B) and fluorescence microscopy (Fig. 6C–E). In these analyses, we observed that the imidazole and EDTA treatments completely or thoroughly disassembled the liposome aggregates which had been induced by membrane-bound Rab5a-His12 proteins (Fig. 6B–E). This indicates that the Rab-mediated membrane aggregation found here is a reversible process of membrane tethering and can be reversibly regulated by membrane attachment and detachment cycles of Rab proteins. Since several prior studies have demonstrated that the stable membrane attachment of Rab proteins is accompanied by GDP/GTP exchange and facilitated by specific Rab guanine nucleotide exchange factors (Ullrich et al., 1994; Soldati et al., 1994; Gerondopoulos et al., 2012; Blümer et al., 2013), the current results lead us to postulate that the GTP requirement for Rab-mediated tethering is directly linked to membrane recruitment of Rab proteins and thereby can be bypassed by artificially membrane-anchored Rab-His12 proteins on DOGS-NTA-bearing membranes in the present chemically-defined system.

Taken together, the current biochemical analyses using purified human Rab proteins and synthetic liposomes have established that membrane-anchored Rab GTPases have the inherent potency to directly mediate reversible membrane tethering events (Figs 3–6). This conclusion is, however, apparently not compatible with the classical membrane tethering model, in which Rab-interacting coiled-coil tethering factors and/or multisubunit tethering complexes, but not Rab GTPases themselves, function as a key component to directly drive membrane tethering (Pfeffer, 1999; Grosshans et al., 2006; Cai et al., 2007; Yu and Hughson, 2010). This study is also not fully consistent with the recent pioneering work by Merz and colleagues, which reported that only yeast endosomal Rabs such as Vps21p, but not the lysosomal/vacuolar Rab GTPase Ypt7p, can support efficient tethering of liposomes (Lo et al., 2012). Our current findings, therefore, reopen the debate about how Rab GTPases, Rab effectors, and tethering factors work together to mediate specific membrane tethering processes in secretory and endocytic membrane trafficking pathways.

## MATERIALS AND METHODS

### Protein purification

The coding sequences of full-length human Rabs (Rab1a, Rab2a, Rab3a, Rab4a, Rab5a, Rab6a, and Rab7a) and HRas proteins were amplified by PCR using the Human Universal QUICK-Clone cDNA II (Clontech) as a template cDNA and cloned into a pET-41 Ek/LIC vector (Novagen) expressing a GST-His6-tagged protein. These PCR fragments contained the sequence encoding the protease cleavage site (Leu–Glu–Val–Leu–Phe–Gln–Gly–Pro) for human rhinovirus 3C protease (Novagen) upstream of the initial ATG codons and the sequence encoding the polyhistidine residues (His12) downstream of the codons for a C-terminal residue, to obtain full-length Rab and HRas proteins with only three extra N-terminal residues (Gly–Pro–Gly) and a C-terminal His12-tag after 3C protease cleavage. To prepare the Rab5a and Rab7a proteins lacking a His12-tag (untagged Rab5a and untagged Rab7a; Fig. 1A, lanes 9 and 10, respectively), the PCR fragments without the His12-coding sequence for these Rab proteins were also amplified and cloned into a pET-41 Ek/LIC vector. Recombinant Rab and HRas proteins were produced in the *Escherichia coli* Rosetta 2(DE3) cells (Novagen) in Terrific Broth medium (1 liter each) with kanamycin (50 µg/ml) and chloramphenicol (50 µg/ml) by induction with 0.5 mM IPTG (34°C, 3 hours). *E. coli* cells were harvested and resuspended in 40 ml each of HN150 (20 mM Hepes-NaOH, pH 7.4, 150 mM NaCl) containing 10% glycerol, 1 mM DTT, 1 mM PMSF, 2.0 µg/ml pepstatin A, and 2 mM EDTA. Cell suspensions were freeze-thawed in a liquid nitrogen bath and a water bath at 25°C, lysed by sonication (UD-201 ultrasonic disrupter; Tomy Seiko, Tokyo, Japan), and centrifuged [50,000 rpm, 75 min, 4°C, 70 Ti rotor (Beckman Coulter)]. GST-His6-3C-tagged Rab and HRas proteins in the supernatants were isolated by mixing with glutathione-Sepharose 4B beads (50% slurry, 2 ml for each; GE Healthcare) and incubating at 4°C for 2 hours with gentle agitation. After washing the protein-bound glutathione-Sepharose 4B beads by HN150 containing 5 mM MgCl_2_ and 1 mM DTT, purified Rab and HRas proteins were cleaved off and eluted by incubating the beads with human rhinovirus 3C protease (12 units/ml final) in the same buffer (2 ml for each protein) at 4°C.

### GTPase activity assay

GTP-hydrolysis activities of recombinant Rab and HRas proteins were assayed by quantitating released free phosphate molecules, using the Malachite Green-based reagent Biomol Green (Enzo Life Sciences). Purified Rab and HRas proteins (4 µM or 6 µM final for each) were incubated at 30°C for 2 hours in HN150 containing MgCl_2_ (6 mM), DTT (1 mM), and GTP (1 mM), GDP (1 mM), or GTPγS (1 mM) where indicated. The reaction mixtures (100 µl each) were then supplemented with 900 µl of the Biomol Green reagent for each, incubated at 30°C for 20 min or 30 min, and analyzed by measuring the absorbance at 650 nm with a DU720 spectrophotometer (Beckman Coulter). The heat-treated Rab and HRas GTPases that had been denatured by treatment at 100°C for 15 min were also tested with the same protocol. Data obtained in this assay were corrected by subtracting the absorbance value of the control reaction assayed in the absence of Rab and HRas proteins. Means and standard deviations of the corrected values (ΔA650) were determined from three independent experiments.

### CD spectroscopy

Far-UV CD spectra of purified recombinant Rab and HRas proteins were measured with a J-820 spectropolarimeter (Jasco) using a cell with a light path of 0.1 mm. Rab1a-His12 (14 µM), Rab2a-His12 (33 µM), Rab3a-His12 (15 µM), Rab4a-His12 (30 µM), Rab5a-His12 (47 µM), Rab6a-His12 (55 µM), Rab7a-His12 (14 µM), HRas-His12 (16 µM), untagged Rab5a (20 µM), and untagged Rab7a (9.3 µM) were analyzed at 4°C in HN150 containing 10% glycerol, 5 mM MgCl_2_, and 1 mM DTT. CD signals obtained at 195–250 nm were expressed as the mean residue ellipticity [*θ*]. Protein secondary structure contents were estimated from CD spectra, using a K2D3 program (Louis-Jeune et al., 2012).

### Liposome preparation

Non-fluorescent lipids were from Avanti Polar Lipids. Fluorescent Rh-PE and fluorescein-PE (FL-PE) were from Molecular Probes. Lipid mixes for the biotin/FL-labeled or Rh-labeled liposomes contained 1-palmitoyl-2-oleoyl (PO) phosphatidylcholine [41% (mol/mol)], POPE (14.5% or 16.5% for the biotin/FL-labeled or Rh-labeled liposomes), soy phosphatidylinositol (10%), PO-phosphatidylserine (5.0%), cholesterol (20%), DOGS-NTA (6.0%), biotin-PE (2.0% for the biotin/FL-labeled liposomes), and fluorescent lipids (1.5% of FL-PE or Rh-PE for the biotin/FL-labeled or Rh-labeled liposomes). Dried lipid films (8 mM lipids) were hydrated in HN150, incubated (37°C, 30 min), freeze-thawed, and extruded 21 times through polycarbonate filters in a mini-extruder (Avanti Polar Lipids) at 40°C. Lipid concentrations were determined from the fluorescence of FL-PE (λex = 495 nm, λem = 520 nm) and Rh-PE (λex = 560 nm, λem = 580 nm).

### Liposome aggregation assay using streptavidin-coated beads

Rab-His12 proteins (4 µM) were mixed with the biotin/FL-labeled (1.8 mM lipids) and Rh-labeled (1.5 mM lipids) liposomes in HN150 containing 6 mM MgCl_2_, 1 mM DTT, and 0.1 mg/ml BSA and incubated with streptavidin-coated beads (Dynabeads M-280 Streptavidin; Invitrogen) (30°C, 2 hours). GTP, GDP, imidazole, EDTA, and a His12 peptide were supplemented where indicated. The streptavidin beads were then washed by HN150 containing 6 mM MgCl_2_ and 1 mM DTT, resuspended in 100 mM β-OG, and centrifuged. To quantify the co-isolated Rh-labeled liposomes, Rh fluorescence of the supernatants was measured by a SpectraMAX Gemini XPS plate reader (Molecular Devices). Means and standard deviations of the Rh fluorescence signals were obtained from three independent experiments.

### Turbidity assay

Turbidity of liposome suspensions was analyzed as described (Ohki et al., 1982). Liposomes (Rh-labeled liposomes, 1.3 mM or 1.0 mM lipids) were mixed with Rabs (0.5–2 µM) in HN150 containing 5 mM MgCl_2_, 1 mM DTT, 0.1 mg/ml BSA, and 2.5% glycerol, followed by measuring the absorbance at 400 nm at room temperature in a DU720 spectrophotometer (Beckman Coulter).

### Fluorescence microscopy

Fluorescence microscopy of liposome suspensions (in HN150 containing 6 mM MgCl_2_, 1 mM DTT, and 0.1 mg/ml BSA) was performed with a BZ-9000 fluorescence microscope (Keyence) equipped with a Plan Apo VC 100×/1.4 NA oil iris objective lens (Nikon), using TRITC and GFP-BP filters (Keyence). Digital images were processed using the BZ-II viewer application (Keyence) and Photoshop CS3 (Adobe).

### Liposome co-sedimentation assay

Rabs (4 µM) were mixed with the Rh-labeled liposomes (1.5 mM lipids) in HN150 containing 6 mM MgCl_2_ and 1 mM DTT, and incubated (30°C, 2 hours). After centrifugation [50,000 rpm, 4°C, 30 min, TLA100 rotor (Beckman)], the pellets and supernatants were analyzed by SDS-PAGE.
